# Plant oil complex: dual activation of peroxisome proliferator-activated receptors α and *γ* to enhance skin barrier function

**DOI:** 10.1515/biol-2025-1347

**Published:** 2026-07-07

**Authors:** Sen Sun, Yanfang Yang, Huijun Wu, Laidi Zhang, Heyang Liu, Lingyan Shen, Hui Xue, Yuankun Liu, Hong Tao

**Affiliations:** Research & Innovation Center, Hangzhou Lanjiang Cosmetics Co., Ltd., Hangzhou 310019, Zhe jiang, China; Research & Innovation Lab, Hangzhou Shiguang Xinya Biotechnology Co., Ltd., Hangzhou 310019, Zhe jiang, China

**Keywords:** skin barrier, peroxisome proliferator-activated receptor α, peroxisome proliferator-activated receptor γ, plant oils, cell culture, genetic analysis

## Abstract

This study aimed to identify novel natural peroxisome proliferator-activated receptor (PPAR) agonists and evaluate the effects of avocado oil unsaponifiable (AOU), *Ganoderma lucidum* spore oil (GLSO), and *Ximenia americana* seed oil (XASO) on skin barrier repair at cellular and human levels, elucidating their combined mechanisms. The oils were extracted using molecular distillation, supercritical CO_2_ extraction, and cold pressing. PPAR activation was assessed via luciferase activity in human embryonic kidney 293 cells (HEK 293T). A blend of the three oils (AGX) was further examined in HaCaT cells using immunofluorescence. A three-dimensional (3D) human skin model was used to evaluate AGX’s effects on barrier-related genes (*FLG*, *LOR, DSG1, TGM1, CLDN1, CER*). A clinical trial measured transepidermal water loss (TEWL) and stratum corneum hydration (SCH). AGX significantly activated *PPARα*-LBD and *PPARγ*-LBD transcription and upregulated *FLG*, *LOR*, *DSG1*, *TGM1*, and *CLDN1* (*p* < 0.01). *In vivo*, both 2 % and 4 % AGX reduced TEWL and increased SCH (*p* < 0.05). AOU and XASO activated PPAR pathway genes (e.g., *ANGPTL4, ILK*) and downregulated *MMP1*. AGX repaired the skin barrier via PPAR pathways and may be a promising therapy for dermatological conditions involving barrier impairment.

## 1 Introduction

Peroxisome proliferator-activated receptors (PPARs) are a class of ligand-activated transcription factors that belong to the superfamily of nuclear receptors (NRs) [1]. Three distinct PPAR isoforms have been identified: *PPARα, PPARγ* and *PPARβ/δ* [2]. All three of these PPARs exhibit distinct patterns of tissue distribution and differ in terms of their ligand-binding domains (LBD) [3], the cavity of the binding domain of PPARα-LBD is similar to PPARγ-LBD, both being 1,400 Å^3^, PPARδ-LBD is smaller, at 1,300 Å^3^ [4]. These isoforms are involved in multiple, distinctive, but often complementary physiological, pathways and functions [5]. Previous research has demonstrated that common skin diseases, such as psoriasis and atopic dermatitis, are characterized by several abnormalities, including the abnormal differentiation of keratinocytes, epidermal hyperplasia, inflammation, and defects in the barrier repair process [1], 2], 6]. The activation of PPARs has been shown to play an important role in the regulation of a variety of skin functions, including the proliferation of keratinocytes, epidermal barrier formation, wound healing, melanocyte proliferation, and the production of sebum [7], 8]. Consequently, PPARs are considered as potential targets for the treatment of various skin diseases [1], 9].

The skin barrier is a vital component for the appropriate functionality of human skin, encompassing essential physical, chemical and biological cutaneous functions. The skin barrier consists of the outermost layer of the epidermis, with the stratum corneum consisting of keratinocytes and intercellular lipids. The maturation of this barrier is accomplished by the coordinated cross-linking of ceramide (CER) and various barrier proteins, including involucrin (IVL), loricrin (LOR), and filaggrin (FLG) via transglutaminase 1 (TGM1) [10]. This highly organized and orchestrated structure protects the skin from dehydration and maintains the skin in a healthy state. These barrier proteins are also known to be regulated by the PPAR pathway [11], [12], [13]. Previous research has been shown that the expression of both *PPARα* and *PPARγ* is reduced in inflammatory skin disorders involving damage to the skin barrier, including hyperproliferative psoriatic epidermis and atopic dermatitis [1], 14], 15]. Consequently, activating the PPAR pathway may serve as a unique means of regulating homeostasis in the skin barrier.

Over recent years, synthetic agonists for *PPARα* and *PPARγ* have been developed as potential drugs for the treatment of metabolic or inflammatory diseases [8], 16]. However, these agonists are associated with several severe side effects, including fluid retention, cardiovascular effects, hepatotoxicity and bone fractures. In contrast, natural ligands, derived from plant oils or natural herbs, can offer practical advantages in terms of safety. The natural ligands for *PPARα* and *PPARγ* are predominantly fatty acids and lipid-derived substrates, including unsaturated fatty acids, eicosanoids and linoleic acid derivatives [17]. For instance, sunflower oleodestillate, a natural product possessing the ability to activate *PPARα*, has been shown to exert beneficial effects on atopic dermatitis in children [18]. A recent study also demonstrated that a plant-derived complex capable of activating *PPARα* could accelerate barrier repair, thus resulting in significant increases in the expression of CER, FLG and TGM1 in skin explant models [19]. Furthermore, a mixture of natural herb extractions was shown to exert dual agonistic effects on both *PPARα* and *PPARγ* and effectively prevented photo-aging when applied topically to photo-aged skin [20].

Natural plant oils contain special components or are enriched in certain rare unsaturated fatty acids that play a vital role in skin barrier repair and anti-inflammation [21]. Historically, avocado oil has sparked a growing interest in human nutrition, the food industry, and cosmetics, and has been associated with cardiovascular system benefits and anti-inflammatory effects [22], 23]. Avocado oil unsaponifiable (AOU), acquired by the molecular distillation of avocado, has been utilized in various anti-inflammatory pharmaceutical and cosmetic preparations [3], 24]. *Ganoderma lucidum* spores are minuscule germ cells ejected from the gills of *G. lucidum*, and contains a diversity of active ingredients, including polysaccharides and triterpenic acid [5]. *G. lucidum* spore oil (GLSO) is extracted from *G. lucidum* spores by treatment with supercritical liquid CO_2_, and contains many triterpenoids, particularly ganoderic acids [25]. A recent study showed that GLSO treatment significantly accelerated the healing of burn wounds on skin of experimental mice thus providing a foundation for the clinical application of GLSO for the treatment of deep skin burns [26]. *Ximenia americana* seed oil (XASO), extracted by the mechanical pressing of *X. americana* seeds, imparts a substantial and smooth consistency to skin care products. Furthermore, XASO also serves as an excellent component for the delivery of long-lasting barrier protection [27], [28], [29].

In this study, we utilized gene activation validation data to design a complex of AOU, GLSO and XASO as a dual activator for *PPARα* and *PPARγ*. By performing *in vivo* and *in vitro* experiments, we investigated the effects of this complex on the expression of key proteins in the epidermal barrier, as well as potential effects on the tissue and composition of lipids in the stratum corneum. We demonstrated, for the first time, that this complex exerted functional effects on human skin barrier repair. Finally, we investigated the potential upstream and downstream signaling pathways responsible for our findings to gain a deeper understanding of the mechanisms underlying the action of complexes derived from vegetable oils on skin.

**Research ethics:** The research related to human use has been complied with all the relevant national regulations, institutional policies and in accordance with the tenets of the Helsinki Declaration, and has been approved by the Ethics Committee of the Lanartisan Skin Health Research Center (ethical review No. 20230104-23-010).

**Informed consent:** Informed consent was obtained from all individuals included in this study, or their legal guardians or wards.

## 2 Materials and methods

### 2.1 Preparation of plant oils

AOU was obtained from virgin avocado oil. Drying of the entire fruit represents an important step in the extraction process as this allows the selective formation of avocadofuran (heptadecadienyl furan). This molecule is then concentrated in two-step molecular distillation process. The final extract contained 30 % unsaponifiable matter, including ≥7 % of avocadofuran. As part of the standard protocol for this study, the temperature for molecular distillation concentration is controlled at 220 ± 40 °C, and the pressure is maintained at 10^−3^ to 10^−2^ mmHg. GLSO was extracted from spores collected from the fruit body. Spores were treated with enzymes released from the spores themselves during different periods of growth. The soluble oil-based fraction was extracted in CO_2_ fluid when the supercritical fluid of CO_2_ was mixed with the treated spores of *G. lucidum*. The final GLSO contained a total triterpene content ≥30 %. The extraction procedure followed the standardized protocol, operating under controlled conditions of 25 ± 1 MPa pressure and 45 ± 5 °C temperature. The supercritical CO_2_ flow rate was maintained at 175 ± 15 L/h, while the separation phase utilized 10 ± 1 MPa pressure and 45 ± 2 °C temperature. XASO was extracted from the kernels of fresh *X. americana* fruit by cold pressing technology. XASO contained ≥20 % of xymenynic acid, a characteristic unsaturated fatty acid.

### 2.2 Cells culture

Human embryonic kidney 293 cells (HEK 293T) were obtained from BeNa Culture Collection (Beijing, China). The turbo chemically competent cells (HaCaT) line was obtained from Beyotime (Shanghai, China). After thawing, these cells were grown in Dulbecco’s modified eagle medium (DMEM) culture medium supplemented with 10 % fetal bovine serum and 100 units/mL of penicillin/streptomycin (all from Gibco, Amarillo, TX, USA) in a humidified CO_2_-incubator under standard conditions (5 % CO_2_, 37 °C). The DMEM medium was changed every three days.

### 2.3 Cell transfection and detection of the luciferase reporter gene

HEK 293T cells were collected at the logarithmic growth phase and transferred to the 6-well plate at a density of 3 × 10^5^ cells/well. Cells were then incubated in a culture chamber at 37 °C and 5 % CO_2_. PcDNA3.1-hPPARαLBD-Gal4DB, pcDNA3.1-hPPARγLBD-Gal4DB or Peak12-6 × Gal4UAS-luci were transfected for 48 h with Lipofectamine 3000 Transfection Reagent in accordance with the manufacturer’s instructions (Thermo Fisher Scientific, Waltham, MA, USA). The mass ratio of pcDNA3.1-hPPARαLBD-Gal4DB or pcDNA3.1-hPPARγLBD-Gal4DB to Peak12-6 × Gal4UAS-luci was 1:1, and their total concentration was 2 μg. Following transfection, cells were further cultured for 24 h to allow for treatment. Cells were transfected for 24 h followed by harvesting and digestion.

Samples included four commercial plant oils (sunflower oil unsaponifiables [SSOU], camellia japonica seed oil [CJSO], meadowfoam seed oil [MSO] and safflower seed oil [SSO], Sanmark Crop, Shanghai, China), three fatty acids (oleic A, linoleic A and linolenic A, Macklin, Shanghai, China), and the AOU, XASO, GLSO, and AGX mixture described above. Approximately 3 × 10^4^ cells per well were then seeded into 96-well plates, and each sample was treated with dimethyl sulfoxide (DMSO), diluted in culture medium to the required concentration (0.1–2.0 %). The control treatment involved DMSO at the same volume ratio. After 24 h of treatment, the medium was discarded, and luciferase activity was measured with a dual-luciferase reporter system (Promega, WI, USA). Luciferase activity served as an indicator of the expression levels of the luciferase reporter gene in cells.

Cells were incubated for 24 h with each compound at a concentration of 10–50 μM and then transfected with the PPARα pZlasmid system (pcDNA3.1-hPPARαLBD-Gal4DB and Peak12-6 × Gal4UAS-luci) and the PPARγ plasmid system (pcDNA3.1-hPPARγLBD-Gal4DB and Peak12-6 × Gal4UAS-luci) top allow comparison between the active substance and the corresponding positive control, fenofibrate and rosiglitazone (MCE, South Brunswick, NJ, USA). DMSO served as a negative control. Luciferase activity was detected and the activation fold-change and relative activity of the compound were calculated. All experiments were performed in triplicate.

### 2.4 Immunofluorescence analysis of PPARα and PPARγ in HaCaT cells

All samples were blocked with 10 % serum for 1 h. Cells were washed twice with phosphate buffer saline (PBS) (Solarbio, Beijing, China) and fixed in PBS containing 4 % paraformaldehyde for 30 min. After two additional PBS washes, cells were permeabilized with PBS containing 0.5 % Triton X-100 for 15 min. Non-specific binding was blocked by incubation in PBS containing 5 % bovine serum albumin (BSA) and 0.1 % Triton X-100 at 37 °C for 30 min, followed by a final PBS rinse. The fixed cells were performed by incubating overnight at 4 °C with PPARα and PPARγ primary antibody (diluted to 1:100) (Abcam, Cambridge, UK). The following morning, sections were washed three times at room temperature (5 min per wash). Then, the sections were incubated at 37 °C with a fluorescein isothiocyanate (FITC)-conjugated secondary IgG antibody (diluted to 1:200) (Abcam, Cambridge, UK) for 2 h. Sections were then stained for 5 min with 4′,6-diamidino-2-phenylindole (DAPI) and observed with a fluorescence microscope (CKX41, Olympus, Tokyo, Japan). For each sample, three random fields of view were analyzed. Data were analyzed with Image Analysis System verion 11.0 software (Crisoptical, Beijing, China).

### 2.5 Histological staining

EpiKutis three-dimensional (3D) human epidermal skins were provided by Guangdong BioCell Biotechnology Co., Ltd. (Guangdong, China), and experiments were conducted using a blank control group, a model group (0.1 % sodium dodecyl sulfonate [SLS]), a positive control group (0.1 % SLS and 50 μM WY14643), and an experimental group (0.1 % SLS and 4 % AGX). The four groups of cells were cultured in an incubator with 5 % CO_2_ at 37 °C for 24 h and then fixed in 40 g/L of paraformaldehyde for 30 min, washed with PBS, dehydrated, embedded in paraffin overnight, sealed with dry gum and cut into 8 μm-thick sections. Sections were then stained with hematoxylin and eosin (HE) and cell morphology was assessed microscopically with an M8 microscope (PreciPoint GmbH, Freising, Germany). For each treatment condition, data were obtained from *n* = 3 independent EpiKutis equivalents.

### 2.6 Immunofluorescence analysis of proteins related to barrier repair in EpiKutis 3D human epidermal skin

EpiKutis 3D human epidermal skin samples were cut into 8 μm-thick sections, incubated in 3 % endogenous peroxidase blocking solution for 10 min and then immersed in normal non-immune serum for 10 min. After blocking non-specific binding, the sections were incubated with FLG, LOR, DSG1, TGM1 and Claudin 1 (CLDN1) primary antibody (Abcam, Cambridge, UK) for 1 h at 4 °C. Thereafter, the sections were treated at 37 °C with biotin-conjugated secondary antibody (diluted to 1:200) (Abcam, Cambridge, UK) and freshly prepared 3,3′ diaminobenzidine (DAB) solution (Santa Clara, CA, USA) and then counterstained with hematoxylin. Finally, immunostained sections were observed under a confocal microscope (C2, Nikon, Tokyo, Japan). For each treatment condition, data were obtained from *n* = 3 independent EpiKutis equivalents.

### 2.7 Ceramide acquisition and qualitative and quantitative detection

The EpiKutis 3D human epidermal skins were treated with different reagents, as described earlier, and then placed in a 2.0 mL centrifuge tube, mixed with protease K solution, and incubated at 50 °C for 2 h. The stratum corneum was then rinsed in deionized water with tweezers and placed in a 2.0 mL centrifuge tube containing lipid extraction solution of chloroform: methanol: water (2:1:1, v/v). Samples were then exposed to ultrasonic shock for 2 min in an ice water bath, transferred to a solution of methanol:chloroform (1:2, v/v), then re-exposed to ultrasonic shock in an ice water bath for 2 min. The entire process was then repeated for a third time. Finally, the extract was dried with nitrogen and re-dissolved with isopropyl alcohol. After centrifugation at 22,000 g for 10 min, the supernatant was aspirated and used for liquid chromatograph mass spectrometer (LC-MS) to determine ceramide levels (Q Exactive, Thermo Fisher Scientific, USA). For each treatment condition, data were obtained from *n* = 3 independent EpiKutis equivalents.

### 2.8 Human study design and outcomes

All test samples were evaluated for safety in human trials. Specific volunteer profiles, treatment methods and processes are described in the Supplementary Material. The skin barrier was evaluated by bioengineering methods (TEWL; Tewameter^®^ TM Hex; Courage & Khazaka, Cologne, Germany) and by stratum corneum hydration (Corneometer^®^ CM 825; Courage & Khazaka, Cologne, Germany) according to the manufacturer’s guidelines [30], 31]. Measurements were repeated three times for each subject area and a mean value was determined.

**Informed consent:** Informed consent was obtained from all individuals included in this study, or their legal guardians or wards.

**Ethical approval:** The research related to human use has been complied with all the relevant national regulations, institutional policies and in accordance with the tenets of the Helsinki Declaration, and has been approved by the Ethics Committee of the Lanartisan Skin Health Research Center (ethical review No. 20230104-23-010).

### 2.9 Transcriptomic profiling and kyoto encyclopedia of genes and genomes (KEGG) analysis

#### 2.9.1 Sample and library preparation

Different samples were co-cultured with HaCaT cells for 24 h and then total RNA was isolated with a RNeasy Mini Kit (TransGen Biotech, Beijing, China). Total RNA was then quantified on a NanoDrop-LITE spectrophotometer (Thermo Fisher Scientific, Waltham, MA, USA). PCR-cDNA sequencing (PCB) libraries were then generated according to the PCR-cDNA sequencing online protocol (SQK-PCB109, ONT, Oxford, UK). cDNA was quantified using a Qubit 4 fluorometer and a Qubit 1X dsDNA HS Assay Kit (Thermo Fisher Scientific, Waltham, MA, USA). CDNA was either used immediately to prepare a sequencing library or stored at −80 °C to await future application.

#### 2.9.2 Transcriptome profiling

A PCR-cDNA Barcoding Kit (Oxford Nanopore Technologies, Oxford, UK) was used to prepare cDNA libraries; all steps were carried out in accordance with the manufacturer’s protocol. The amplified and barcoded cDNA samples (100 fmol in total) were pooled together to a final volume of 11 μL in Elution Buffer, which was then used for sequencing [32]. Nanopore sequencing was carried out on a MinION sequencer (Oxford Nanopore Technology, Oxford, UK) in 72 h single runs, using FLO-MIN106D flow cells and PCR-cDNA sequencing-barcoding (SQK-PCB109, ONT, Oxford, UK). The sequencing libraries were prepared in accordance with the manufacturer’s protocol and sequenced immediately. Outputs varied between 300 and 900 Mb. Raw sequencing data for both platforms were deposited in the NCBI SRA (SRR36222951, SRR36222952, SRR36222953; https://www.ncbi.nlm.nih.gov/).

#### 2.9.3 KEGG analysis

Nanoplots were used for off-machine data quality control. Quality control for ONT data followed the epi2me “Basic QC” pipeline (https://labs.epi2me.io/). ONT fastq files were aligned using the long-read aligner Minimap2 using the “ax splice” mode [33]. Salmon software was used to compare and quantify the sorted files to acquire transcriptome expression data. Differential expression analysis for two conditions/groups was performed using the DESeq2 R package (1.42.0) [34]. DESeq2 provides statistical programs for determining differential expression in digital gene expression data using models based on negative binomial distribution. The resulting *p*-value is adjusted using the Benjamini and Hochberg’s methods to control the error discovery rate. The threshold of significant differential expression: padj ≤ 0.05 & |log2(foldchange)| ≥ 1. Up- and down-regulated differentially expressed genes (DEGs) were subjected to KEGG pathway enrichment analysis (DAVID bioinformatics resources), by the clusterProfiler R package (4.8.1) was implemented Enrichment was considered significant at *p* < 0.05 (Benjamini-Hochberg corrected). Based on the annotation results, the protein-protein interaction network (PPI) was constructed by combining with the STRING database (https://cn.string-db.org/), and the interrelationships among the genes were analyzed.

### 2.10 Statistical analysis

Data are expressed as means ± standard deviation (SD). Statistical analysis was conducted using GraphPad Prism version 9.0 (GraphPad Software, San Diego, CA, USA). The Student’s *t*-test was used to determine the significance of differences at *p* < 0.05 (*), *p* < 0.01 (**) and *p* < 0.001 (***) for *in vitro* experiments. In the human study, we used the Shapiro-Wilk test to assess data normality; this was followed by paired *t*-tests or rank-sum Wilcoxon paired tests between target samples and controls. All tests were bilateral with an *α* = 0.05. Statistical significance in the human study was determined by analysis of variance (ANOVA) and *p* < 0.05 (*) was considered statistically significant.

## 3 Results

### 3.1 Activation of PPARα and PPARγ by plant oils

**Figure 1:**
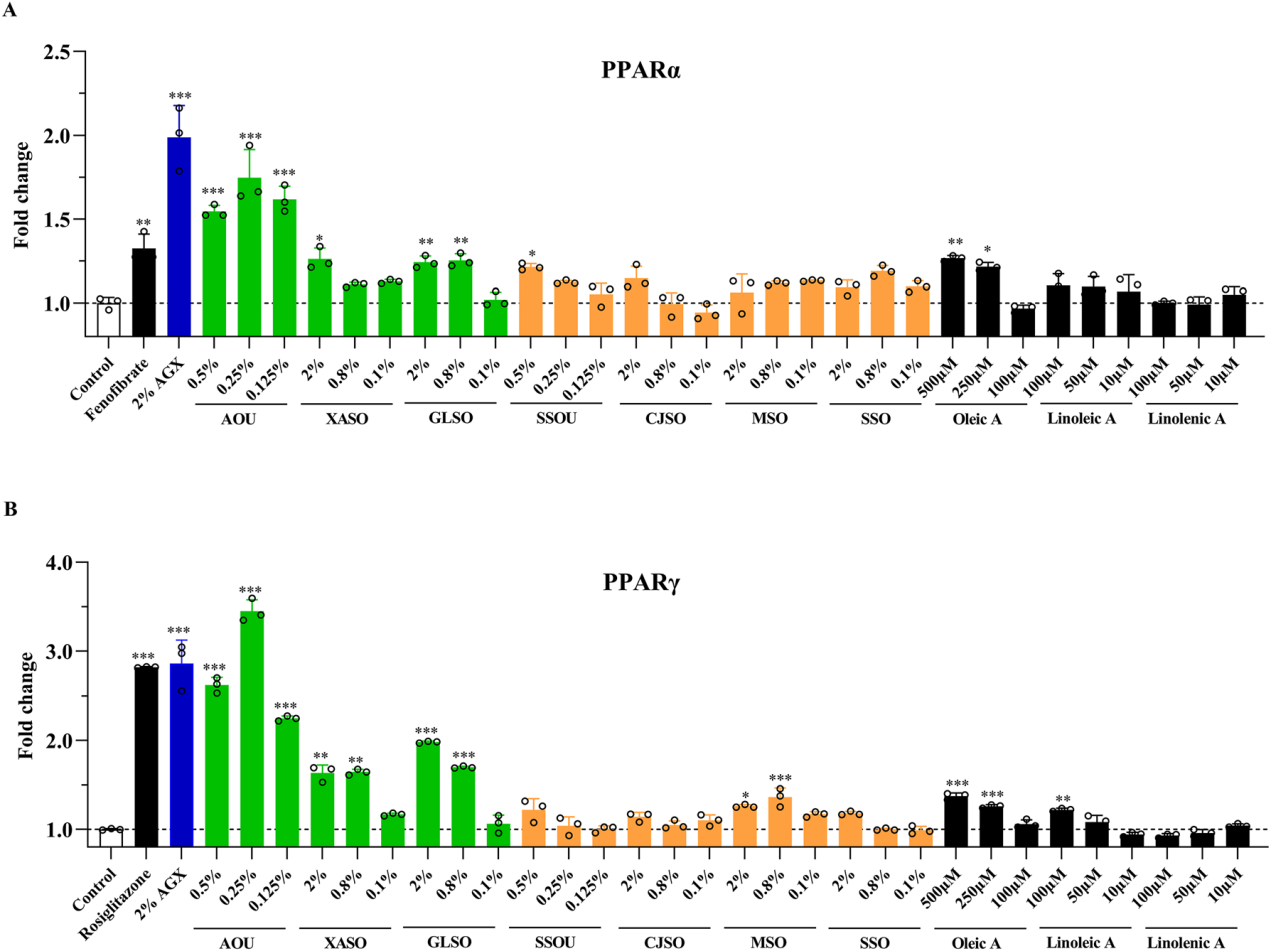
Effect of different plant oils and free fatty acids on the transcriptional activities of PPARα-LBD and PPARγ-LBD. (A) Fold change of PPARα-LBD activation, as determined by luciferase reporter assays; (B) fold change of PPARγ-LBD activation, as determined by luciferase reporter assays. Fenofibrate and rosiglitazone (10 μM) were used as respective positive controls. All data are expressed as mean ± SD (*n* = 3). PPARα, peroxisome proliferator-activated receptors α; PPARγ, peroxisome proliferator-activated receptors γ; AOU, avocado oil unsaponifiable; GLSO, *Ganoderma lucidum* spore oil; XASO, *Ximenia americana* seed oil; SSOU, sunflower seed oil unsaponifiable; CJSO, *Camellia japonica* seed oil; MSO, meadowfoam seed oil; SSO, safflower seed oil; AGX, complex composed of AOU, GLSO and XASO. The Student’s *t*-test was used to determine the significance of differences at *p < 0.05, **p < 0.01 and ***p < 0.001 for the experiments.

To verify the effect of AOU, GLSO and XASO on PPAR activation, a range of concentrations were selected for each of the plant oils. The positive control (fenofibrate; 10 μM) increased *PPARα*-LBD expression by 32.41 % (Figure 1A), while the other positive control (rosiglitazone; 10 μM) enhanced the expression of *PPARγ*-LBD by 181.96 % (Figure 1B). All three concentrations of AOU significantly activated both *PPARα*-LBD and *PPARγ*-LBD (*p* < 0.001). Notably, the 0.25 % concentration of AOU increased the expression of *PPARα*-LBD and *PPARγ*-LBD by 32.02 % and 22.34 % higher than fenofibrate and rosiglitazone, respectively (Figure 1A and B). Compared with controls, 2 % XASO significantly activated *PPARα*-LBD *and PPARγ*-LBD by 126.15 % and 163.27 %, while 0.8 % XASO activated *PPARγ*-LBD by 164.37 % (Figure 1B). Moreover, 2 % and 0.8 % GLSO significantly activated *PPARα*-LBD and *PPARγ*-LBD (*p* < 0.01) (Figure 1B). Of the 10 oil treatments tested AOU, GLSO and XASO effectively activated the expression of *PPARα*-LBD *and PPARγ*-LBD as ligands (Figure 1A and B). Thus, the combination of AOU, GLSO and XASO was selected to generate a skin repair complex (hereafter referred to as AGX). Next, we compared 2 % AGX (composed of 0.125 % AOU, 1 % GLSO and 0.875 % XASO) and individual plant oils or unsaturated fatty acid. Compared with SSOU, CJSO, MSO, SSO, oleic acid, linoleic acid, and linolenic acid, 2 % AGX exhibited stronger ability to activate PPARα-LBD and PPARγ-LBD (*p* < 0.001) (Figure 1A and B).

### 3.2 A complex derived from plant oils upregulated the expression of PPARα and PPARγ proteins

**Figure 2:**
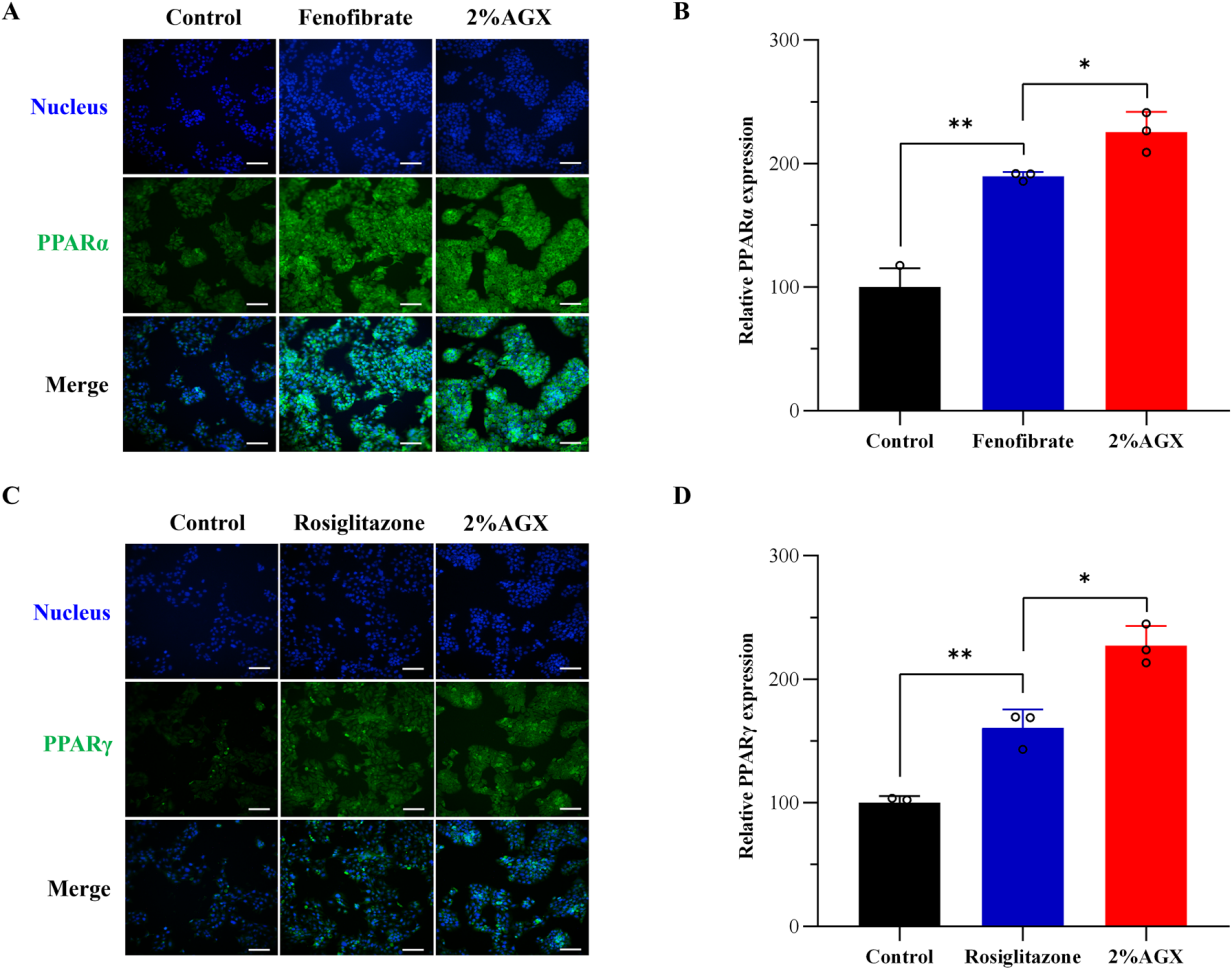
The AGX complex, derived from plant oils, increased the expression of PPAR protein in HaCaT cells. Fenofibrate and rosiglitazone (10 μM) were used as positive controls in the experiment. (A) Images of PPARα immunofluorescence staining (×100) in HaCaT cells. Green fluorescence represents PPARα while blue fluorescence represents the nuclei; (B) quantitative analysis of PPARα immunofluorescence images, as performed by Image Analysis System 11.0 software; (C) images of PPARγ immunofluorescence staining (×100) in HaCaT cells. Green fluorescence represents PPARγ while blue fluorescence represents the nuclei; (D) quantitative analysis of PPARγ immunofluorescence images, as performed by Image Analysis System 11.0 software. Scale bar represents 100 µm. All data are expressed as mean ± SD (*n* = 3). PPAR, peroxisome proliferator-activated receptors; AGX, complex composed of avocado oil unsaponifiable, *Ganoderma lucidum* spore oil and *Ximenia americana* seed oil. The Student’s *t*-test was used to determine the significance of differences at *p < 0.05, **p < 0.01 and ***p < 0.001 for the experiments.

Immunofluorescence is frequently used to localize specific proteins of interest and is commonly used to evaluate how different experimental treatments can impact protein expression. Here, we evaluated the effects of 2 % AGX complex on PPARα and PPARγ protein expression in human keratinocytes (HaCaT cells) by immunofluorescence. Compared with controls, the expression levels of PPARα and PPARγ were significantly increased after 24 h of culturing HaCaT cells with 2 % AGX (*p* < 0.01) (Figure 2A and C). Moreover, the expression levels of PPARα and PPARγ protein in the presence of AGX were higher than the positive controls (*p* < 0.05), fenofibrate and rosiglitazone (Figure 2A and C). PPARα protein expression increased by 189.7 % in response to fenofibrate and increased by 225.7 % in response to AGX treatment (*p* < 0.01) (Figure 2B). Compared to the positive control, the expression PPARα protein increased by 119.0 % in response to AGX treatment (*p* < 0.05). PPARγ protein expression increased to 160.5 % in response to rosiglitazone treatment and by 227.3 % in response to the AGX treatment (*p* < 0.01) (Figure 2D). Compared to the positive control, the expression of PPARγ protein increased by 141.6 % in response to the AGX treatment (*p* < 0.05). Based on the relative quantitative data, our findings revealed a trend toward increased expression of PPARα and PPARγ proteins following treatment with the plant oil complex (AGX). A comparative analysis suggested that the magnitude of this increase was greater than that observed with the positive controls, fenofibrate and rosiglitazone.

### 3.3 The AGX complex, derived from plant oils improved epidermal morphology and stimulated barrier repair-related protein expression *in vitro* reconstructed skin model

**Figure 3:**
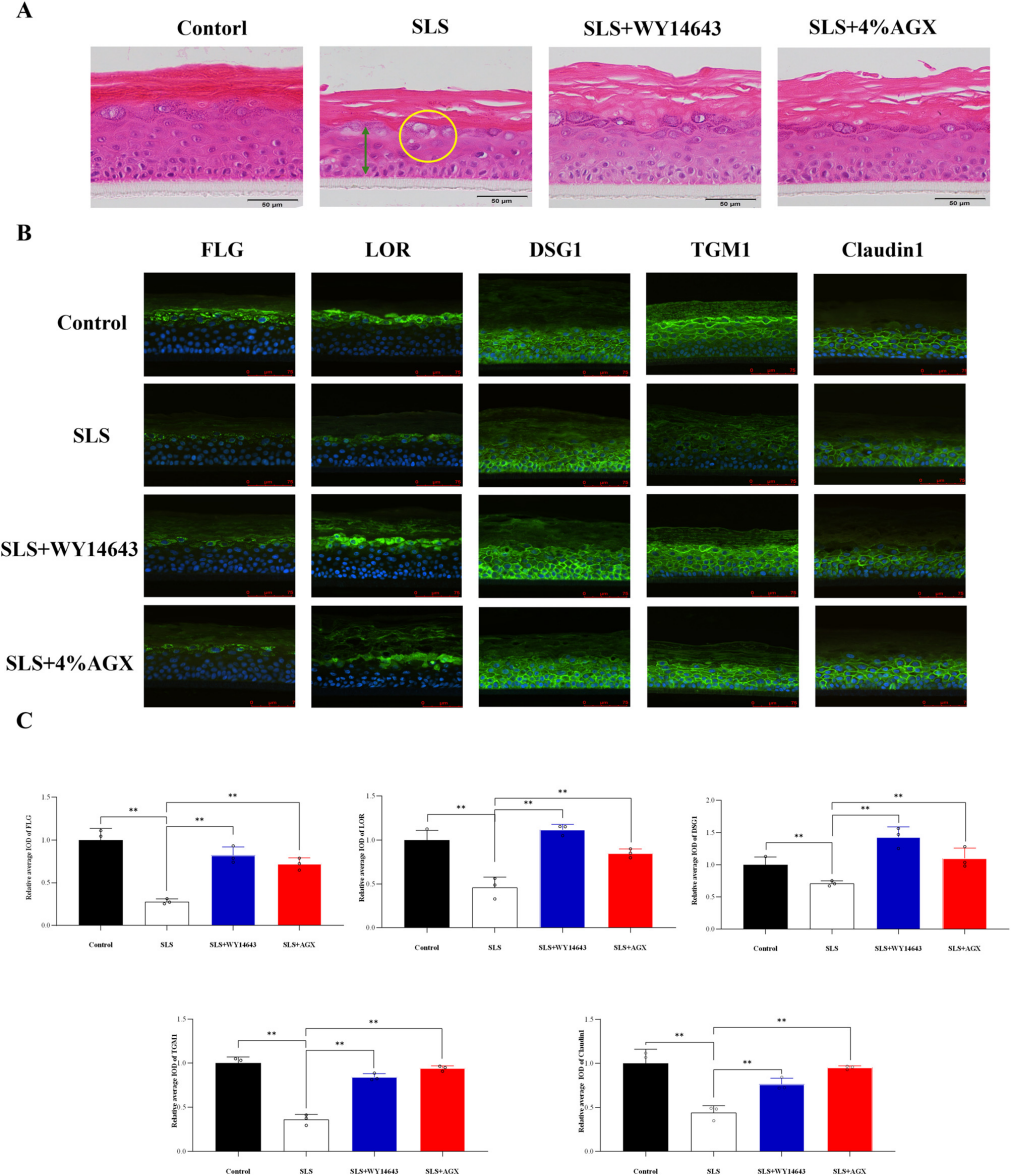
The protective and healing effects of a complex derived from plant oils on an SLS-induced EpiKutis 3D human skin damage model. (A) H&E staining in the epidermal explant model; red-stained areas correspond to keratin and blue-purple-stained areas correspond to nucleic acids. Vacuoles and damage to the living cell layer are marked with a yellow box in the SLS-treated negative model group; (B) FLG, LOR, DSG1, TGM1 and Claudin1 immunofluorescence staining (×100) in the epidermal explant model; (C) the relative expression levels of FLG, LOR, DSG1, TGM1 and Claudin1 proteins. All data are expressed as mean ± SD (*n* = 3). AGX, complex composed of avocado oil unsaponifiable, *Ganoderma lucidum* spore oil and *Ximenia americana* seed oil; SLS, sodium dodecyl sulfonate; FLG, filaggrin; LOR, oricrin; DSG1, desmoglein 1; TGM1, transglutaminase 1. The Student’s *t*-test was used to determine the significance of differences at *p < 0.05, **p < 0.01 and ***p < 0.001 for the experiments.

To investigate the protective and reparative effects of AGX on the recovery of a damaged epidermal barrier, we utilized an epidermal explant model (EpiKutis^®^3D human skin) that had been exposed to SLS (0.1 %, v/v) irritation, followed by the topical application of 4 % AGX. H&E results showed that in the SLS-induced damage model, the stratum corneum of the epidermal layer exhibited a loose structure, the living keratinocyte layer was damaged and reduced in thickness, vacuoles were evident, and notable damage was observed compared to the control (Figure 3A). WY14643 (50 μM), a PPARα agonist, was utilized as the positive control in the experiment to restore homeostasis and improve skin barrier function in the deficient skin models by normalizing the free fatty acid profile [35]. Compared with the SLS-treatment, under the positive control condition, the boundaries between the four layers of the model were clearer, living keratinocyte were arranged in a more compact manner, and the damage of the activated keratinocyte layer and the size of the vacuoles that appeared were significantly reduced. For 4 % AGX, the observed effects were similar to the corresponding effects observed in the positive control, thus indicating that skin barrier function had been improved by AGX (Figure 3A).

Next, we used the SLS-induced model of epidermal damage to investigate the expression levels of proteins related to barrier repair (FLG, LOR, DSG1, TGM1 and CLDN1) by immunofluorescence staining. Analysis indicated that the expression levels of FLG, LOR, DSG1, TGM1 and CLDN1 in the SLS treatment were significantly lower than those in the control treatment (*p* < 0.01). Furthermore, levels of these proteins were significantly increased in the positive treatment and the 4 % AGX treatment (*p* < 0.01), as compared to the SLS treatment (Figure 3B and C). These data suggested that 4 % AGX protected SLS-induced EpiKutis 3D human epidermal skin from damage by upregulating the expression of FLG, LOR, DSG1, TGM1 and CLDN1 proteins.

**Figure 4:**
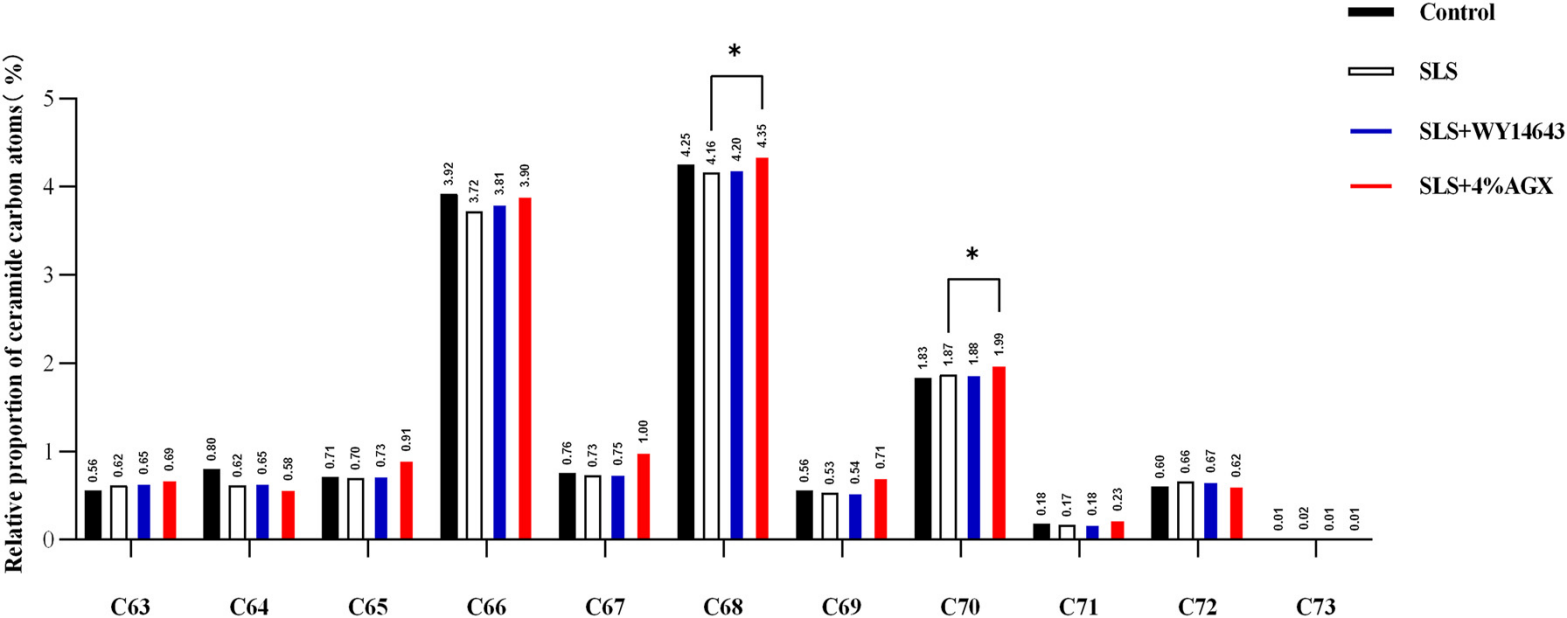
The relative proportion of different long-chain ceramides (C63–C73) in different treatment groups. All data are expressed as mean ± SD (*n* = 3). AGX, complex composed of avocado oil unsaponifiable, *Ganoderma lucidum* spore oil and *Ximenia americana* seed oil; SLS, sodium dodecyl sulfonate. The Student’s *t*-test was used to determine the significance of differences at *p < 0.05, **p < 0.01 and ***p < 0.001 for the experiments.

Previous studies have shown that the shortening of CER carbon chains can aggravate water loss from the skin, and the impact on the barrier function was much greater than that caused by a change in the subclass of CER [36]; consequently, this represents an important index with which to evaluate skin barrier function. Thus, the long-chain CER carbon chains in an SLS-induced model of damage treated with 4 % AGX were qualitatively and quantitatively determined by LC-MS. Compared with SLS treatment, the 4 % AGX treatment increased the relative proportions of long-chain CER C63, C65, C66, C67, C68, C69, C70 and C71 by 11.29 %, 30.00 %, 4.84 %, 36.99 %, 4.57 %, 33.96 %, 6.42 % and 35.29 %, respectively. In particular, the relative proportion of C68 and C70 increased significantly (*p* < 0.05) (Figure 4). Collectively, these data suggested that 4 % AGX may increase the content of long-chain CERs in the damaged stratum corneum to enhance skin barrier function.

### 3.4 A complex derived from plant oils enhanced skin barrier repair *in vivo*

**Figure 5:**
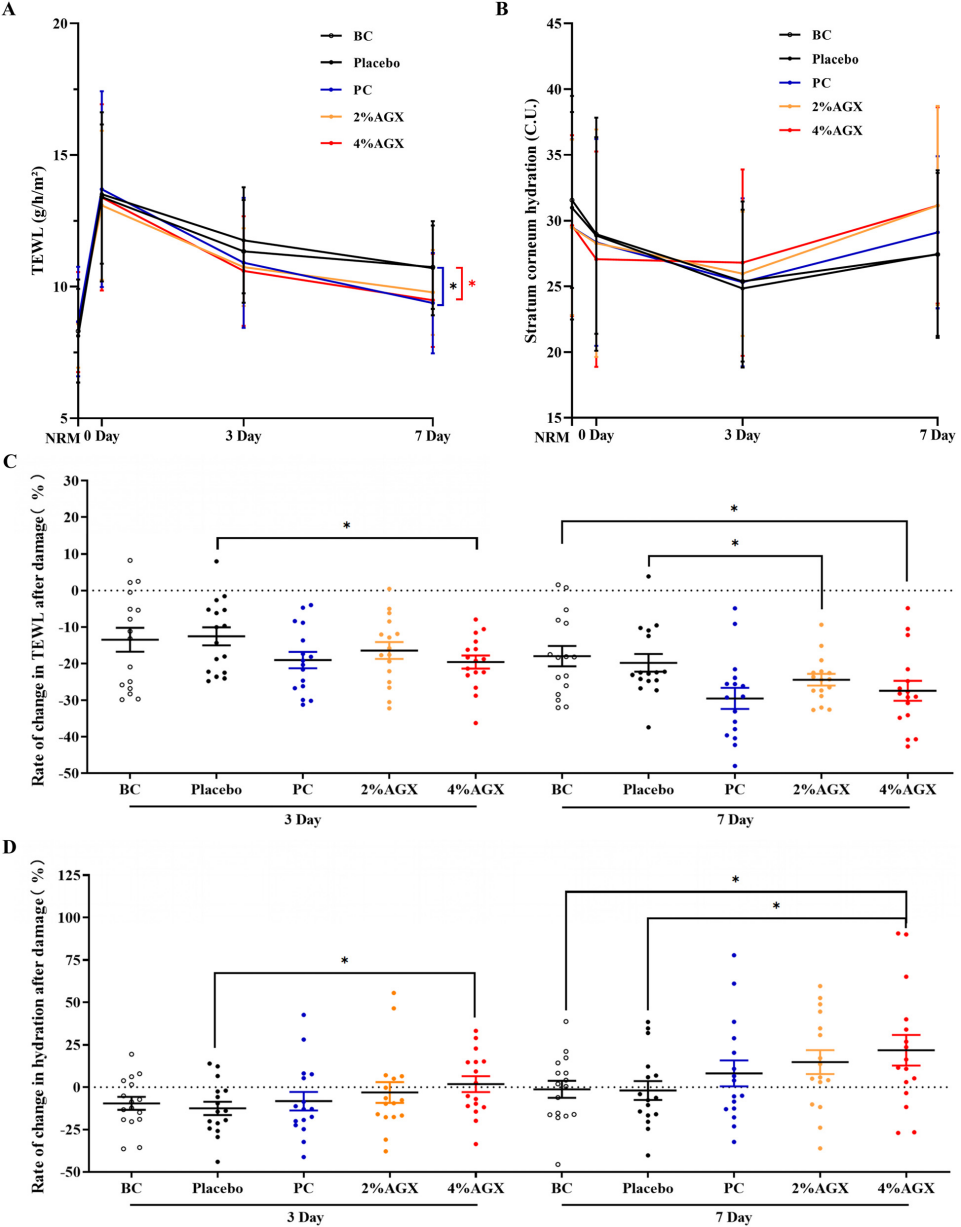
Changes in the TEWL and stratum corneum hydration following treatment with a complex derived from plant oils in SLS-irritated human skin. (A) Changes in TEWL (g/h/m^2^) before irritation, after irritation without any treatment for three days, after treatment or three days and after treatment for 7 days; (B) changes in stratum corneum hydration (C.U.) before irritation, after irritation without any treatment for three days, after treatment for three days and after treatment for 7 days; (C) rates of change in the TEWL after damage with treatment for three days and seven days; (D) rates of change in stratum corneum hydration after damage with treatment for three days and seven days. All data are expressed as mean ± SD (*n* = 16). NRM, normal skin before irritation; 0 day, skin after irritation without any treatment for three days; 3 day, skin after treatment for three days; 7 day, skin after treatment for 7 days; BC, blank control group; PC, positive control group; TEWL, trans-epidermal water loss. Data normality was verified with the Shapiro-Wilk test. Inter-group comparisons (target vs. control) were performed using paired *t*-tests or Wilcoxon signed-rank tests, as appropriate. Significance was determined by ANOVA with *p < 0.05 considered statistically significant.

To confirm the effects of the AGX complex on an impaired human skin barrier, we conducted a single-center and double-blinded trial. Exposure to SLS irritants can induce clinically acute barrier damage in the skin, resulting in symptoms such as skin dryness, an increase in trans-epidermal water loss (TEWL), and a reduction in stratum corneum hydration. At baseline, the average TEWL ranged from 8.14 ± 1.78 to 8.67 ± 2.08 g/h/m^2^, and the mean stratum corneum hydration in different test areas ranged from 29.5 ± 6.7 to 31.6 ± 6.7 corneometer unit (C.U). After 24 h pre-treatment with 1 % SLS, the Finn Chamber was removed without any further treatment for 3 days. After three days, the mean TEWL significantly increased to a range of 13.09 ± 2.83 to 13.71 ± 3.73 g/h/m^2^ (*p* < 0.001) (Figure 5A), and the average stratum corneum hydration decreased to a range of 27.1 ± 8.2 to 29.0 ± 8.9 C.U. (Figure 5B), overall, data indicated the successful induction of barrier damage.

After the plant oil complexes (2 % and 4 % AGX), placebo and positive drugs were applied twice a day for three days, compared with the placebo group, the rate of change in the TEWL after damage was significantly reduced in the 4 % AGX group (−19.6 % vs. −12.6 %, *p < *0.05). Following seven days of treatment, compared with the blank and placebo groups, the rate of change in the TEWL after damage was significantly reduced in the 4 % AGX group (−27.4 % vs. −18.0 %, *p* < 0.05; −27.4 % vs. −19.8 %, *p* < 0.05). Furthermore, the rate of change in the TEWL after damage was also significantly reduced in the 2 % AGX group when compared with placebo groups (−24.4 % vs. −19.8 %, *p* < 0.05) (Figure 5C). Moreover, in terms of stratum corneum hydration, similar improvement was also evident in the 4 % AGX group. When applied twice a day for three days and seven days, compared with placebo group, the rate of change in hydration following damage was significantly increased in the 4 % AGX group (1.8 % vs. −12.6 %, *p* < 0.05; 21.7 % vs. −2.0 %, *p* < 0.05) (Figure 5D). In summary, these results suggest that AGX, at concentrations of 2 % and 4 %, has the potential to enhance skin barrier repair, providing preliminary evidence for its bioactivity.

### 3.5 The mechanism underlying the effects of plant oil extracts on skin barrier repair as determined by PPAR pathway analysis

**Figure 6:**
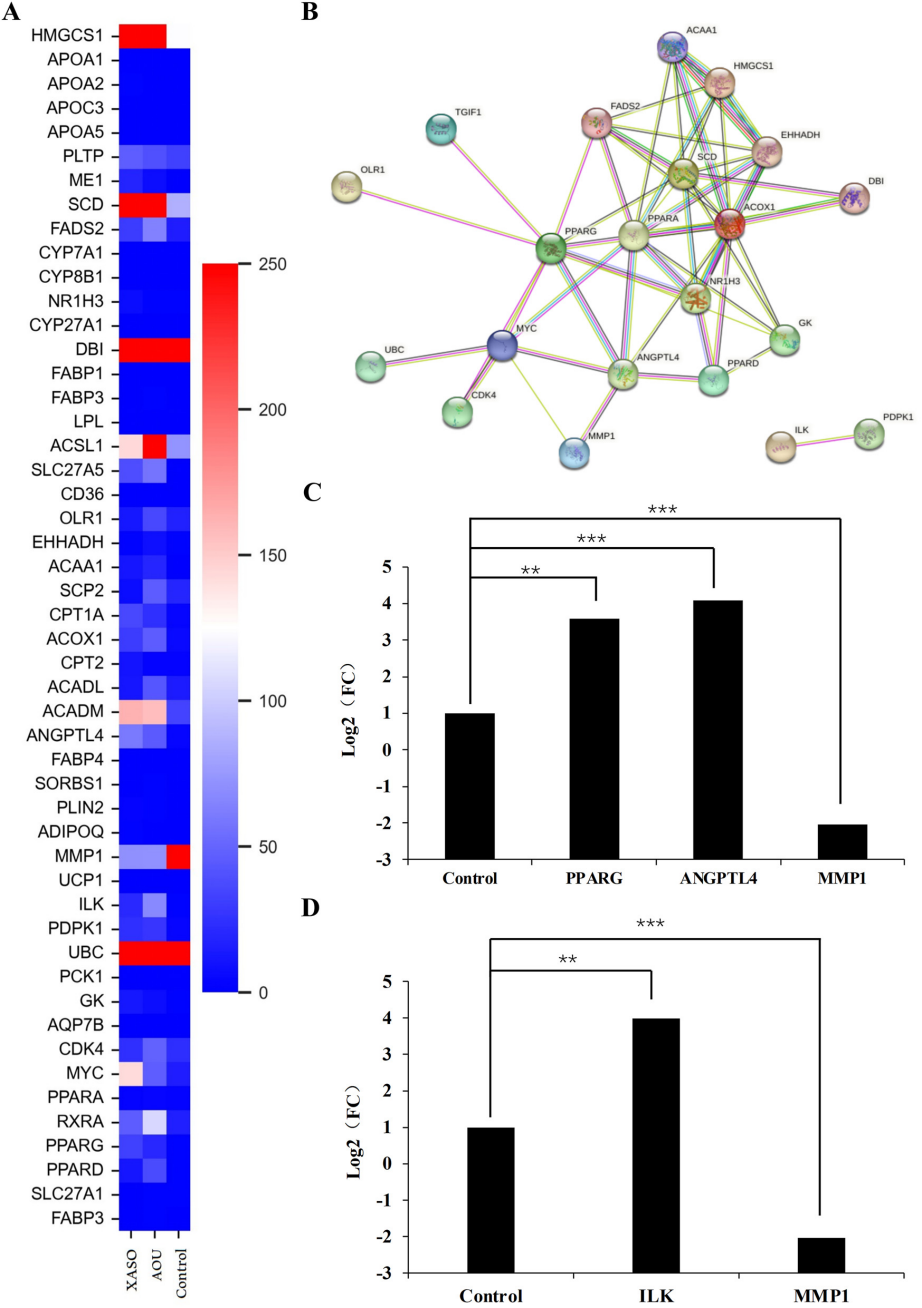
KEGG pathway enrichment analysis of the integrated DEGs. (A) Heat map of DEGs enriched in the PPAR pathway. Expression values are presented as count values. Red colors indicate upregulated DEGs while blue colors indicate downregulated DEGs (XASO, cells treated with 2.5 % XASO for 24 h; AOU, cells treated with 1.25 % AOU for 24 h; Con, cells without any treatment). The scale bar represents expression values; (B) PPI network analysis of 21 DEGs downstream of the PPAR pathway, as identified by the STRING database; (C) the effects of XASO on genes downstream of the PPAR signal pathway; (D) the effects of AOU on genes downstream of the PPAR signal pathway. All data are expressed as mean ± SD (*n* = 3). XASO, *Ximenia americana* seed oil; AOU, avocado oil unsaponifiable; KEGG, kyoto encyclopedia of genes and genomes; DEGs, differentially expressed genes; PPAR, peroxisome proliferator-activated receptor; PPI, protein-protein interaction network; ILK, integrin-linked kinase; MMP1, matrix metalloproteinase 1; ANGPTL4, angiopoietin like 4. The resulting *p*-value is adjusted using the Benjamini and Hochberg’s methods to control the error discovery rate. **p < 0.01, ***p < 0.001.

To elucidate the mechanism by which plant oils promote skin barrier repair, we next performed PPAR pathway analysis. KEGG-based pathway analysis was carried out to collect information relating to protein functionality in metabolic processes to identify the specific biological events DEGs [37]. We focused on the roles of AOU and XASO which had been shown to significantly activate *PPARα* and *PPARγ* transcription in the PPAR signaling pathway. We identified a total of 50 enriched genes in the PPAR pathway, as shown in a heat map (Figure 6A). Compared to the control, nine genes in the PPAR signal pathway (*RXRA*, *MYC*, *MMP1*, *ANGPTL4*, *ACADM*, *SLC27A5*, *ACSL1*, *SCD* and *HMGCS1*) were regulated by AOU and GLSO treatment.

Although the heat map revealed the entire set of genes in the PPAR signaling pathway, we focused on whether AOU and XASO could activate genes downstream of the PPAR pathway. Therefore, a PPI network analysis of the 21 DEGs based on the STRING database were established in order to further understand the functionality of the PPAR pathway and demonstrate how AOU and XASO activated this pathway. The PPI network included 21 nodes (Figure 6B). The top 10 genes in the network ranked by degree method were *PPARA, PPARG, HMGCS1, ACOX1, SCD, ANGPTL4, MYC, FAPS2, MMP1 and CDK4* (Figure 6B); these represented key molecules of the canonical PPAR pathway.

Of these top 10 genes, *ANGPTL4* is one of the main paralogous genes for both *PPARγ* and *PPARα.* Analysis showed that XASO could promote the expression of *ANGPTL4* when compared with the control (*p* < 0.001) (Figure 6C). Moreover, matrix metallopeptidase 1 (*MMP1*), one of the main paralogous genes of *ANGPTL4* (Figure 6B), was downregulated in HaCaT cells when treated with AOU and XASO (Figure 6C and D). Notably, XASO directly increased the expression of *PPARγ* when compared with the control (*p* < 0.001) (Figure 6C). AOU promoted the expression of integrin-linked kinase (*ILK*) (*p* < 0.01) which is known to contribute to epidermal integrity and the establishment of barrier properties [38] (Figure 6D).

In summary, we hypothesize that AOU and XASO can regulate the skin barrier and specifically modulate the PPAR signaling pathway by up-regulating the expression of *PPARγ*, *ANGPTL4* and *ILK* in HaCaT cells, and by down-regulating *MMP1*.

## 4 Discussion

PPARs are a class of transcription factor that multi-directionally regulate the participation of macrophages in each stage of skin barrier repair. PPARα and PPARγ are involved in regulating skin repair in the early inflammatory stage and the transcription of inflammatory mediators, and are involved in almost every step of skin barrier repair. It has been proven that vegetable oils contain natural receptors for PPAR and are associated with a high safety level [21].

Recent advancements have further clarified the complex role of PPARs in skin barrier homeostasis and the therapeutic potential of natural ligands. Studies have confirmed that the activation of PPARα not only regulates lipid metabolism but also directly influences the differentiation program of keratinocytes, thereby enhancing the expression of terminal differentiation markers such as filaggrin and keratin-like proteins [39]. At the same time, PPARγ has been identified as a key regulator of epidermal inflammation and lipid synthesis in the stratum corneum; its activation can restore the lipid lamellar structure in atopic dermatitis models [40]. It is notable that the synergistic effect between PPARα and PPARγ activation has been emphasized as a superior comprehensive barrier repair strategy compared to single-pathway targeting, as it simultaneously addresses the issues of differentiation, lipid production, and anti-inflammatory response [41]. Our findings indicate that the role of the AGX complex as a dual activator is fully consistent with this emerging model. Our research results indicate that the use of AGX in acute SLS damage models can rapidly repair the barrier, which expands these clinical observations and provides a mechanistic basis for its efficacy through dual receptor activation and downstream gene regulation (such as *ANGPTL4*, *ILK*).

In the search for ligands derived from plant oils with the dual ability to activate PPARα and PPARγ, we conducted dual luciferase reporter assays, resulting in the identification of three potential plant oils, AOU, GLSO and XASO. Of these, AOU was mainly composed of avocadofuran, represented by heptadecadienyl furan, which is known to be useful as an anti-fibrotic drug for the treatment of diseases involving excess collagen deposition [42], 43]. It is generally accepted that the pharmacological action of GLSO is closely associated with its e nriched content of rare Ganoderma triterpenes. A recent study reported that the topical application of GLSO may downregulate inflammation by regulating the skin microbiota to accelerate skin wound healing [44]. XASO is particularly rich in ximenynic acid, an acetylene fatty acid, which has been investigated for a variety of skin protective and anti-inflammatory applications [19], 45]. In the present study, we showed AOU, GLSO and XASO exerted significant and efficient dual activation of the transcriptional activities of *PPARα*-LBD and *PPARγ*-LBD genes when compared with some commonly used plant oils and unsaturated fatty acids which are known to activate *PPARα*-LBD and *PPARγ*-LBD genes. In particular, AOU exhibited the strongest activation effect, even at low concentrations. According to the action concentrations of the three plant oils and the corresponding activation effects, we next conducted comprehensive concentration-utility ratio analysis and screened the combination of 2 % AGX complex (composed of 0.125 % AOU, 1 % GLSO and 0.875 % XASO). Analysis revealed that this complex not only significantly activated *PPARα*-LBD and *PPARγ*-LBD gene transcription, but also exerted a synergistic effect when compared to individual plant oils.

PPARα and PPARγ may serve as unique regulators of barrier homeostasis by normalizing the skin lipid ratio and increasing the expression of FLG and other structural proteins [34], 46], 47]. Thus, we established a epidermal explant model of skin damage, which was used to verify the effects of the AGX complex on epidermal structure, proteins related to the skin barrier, and CERs in stratum corneum lipids *in vitro*. We found that AGX significantly improved keratinocyte vacuolation and upregulated the expression of FLG, LOR, DSG1, TGM1 and CLDN1 proteins. During the formation of the skin barrier, these cell membrane proteins in the stratum corneum are extensively cross-linked to form the cuticular envelope (CE). The CE is composed of FLG and LOR, and creates a ‘brick-like’ structure in the skin barrier. Interestingly, the presence of TGM1 in the epidermis is involved in the formation of the CE [48]. In addition, desmosomes are one of the important intercellular links in the mechanical integrity of the epidermis. Of these, DSG1 is a key transmembrane glycoprotein in desmosomes and helps keratinocytes to enhance stability and reduce mechanical damage [49]. Outside of the stratum corneum, the epidermal tight junction (TJ) is also an essential structure for the integrity of the skin barrier, and is predominantly composed of claudins and occludin proteins [50]. The membrane expression of TJ proteins is closely related to the physical defense functionality of the barrier, and a variety of TJ proteins interconnect to form the TJ complex, which connects cells to each other or to the extracellular matrix [51].

When analyzing skin lipid metabolomics, we mainly focused on CERs which account for up to 50 % of total lipid content [52]. CER content and chain length are known to be critical for the lipid bilayer structure and skin barrier functionality. Our analysis revealed that AGX increased the content of long-chain CERs in the damaged stratum corneum to enhance skin barrier function. It has been previously demonstrated that shortening of CER chain length can exacerbate water loss from the skin surface; furthermore, the effect on barrier function was much greater than that caused by changes in CER subclasses [35].

Further, the outcomes of our preliminary studies on human skin offer initial clinical insights into the potential benefits of the plant oil-derived complex AGX for skin barrier repair. The observed reduction in TEWL and increase in stratum corneum hydration suggest a potential role for AGX in improving skin barrier parameters. While our study highlights the potential benefits of AGX, further research is warranted to assess its long-term effects and to validate these initial observations. Large-scale clinical trials are also required to confirm the efficacy of AGX in diverse populations and dermatological conditions.

In addition, investigating the specific molecular pathways by which AGX activates PPARα and PPARγ could provide a deeper understanding of its mechanism of action. We investigated how two representative plant oils (AOU and XASO) might regulate the PPAR signaling pathway. We found that AOU and XASO can regulate the PPAR signaling pathway by up-regulating *ANGPTL4* and *ILK*, and by down-regulating *MMP1*, thus ultimately playing a role in skin barrier repair. *ANGPTL4* can promote the proliferation and migration of epidermal stem cells and can contribute to cutaneous wound re-epithelialization by up-regulating the expression of cyclins D1 and A2 and by accelerating cell cycle transition from G1 to S phase [53]. *ILK* is also an essential component of keratinocyte differentiation programs that contribute to epidermal integrity and the establishment of barrier properties [54]. In the absence of *ILK*, the calcium sensing receptor, E-cadherin, and Zona Occludens 1 (ZO-1) fail to translocate to the cell membrane; this occurs via mechanisms associated with abnormalities in microtubules and RhoA activation. Moreover, as one of main paralogous genes of *ANGPTL4*, *MMP1* is a member of the zinc-dependent family of enzymes [55], which can specifically degrade proteins in connective tissues, such as collagen I. When overexpressed, *MMP1* specifically degrades the extracellular matrix, destroys the normal structure of collagen and elastic fibers, and leads to wrinkles or other skin aging manifestations. AOU and XASO may inhibit *MMP1* activity and abnormal collagen degradation, which is important for delaying aging.

This study has several limitations that should be considered. First, the preliminary screening for PPARα/γ transcriptional activation in this study was conducted in HEK 293T cells. Although this cell line shows high transfection efficiency and yields stable and reliable results in luciferase reporter assays, the reporter system employed is not derived from skin cells and cannot fully recapitulate the full-length PPAR transcriptional regulatory program mediated by endogenous peroxisome proliferator response elements (PPREs) in skin cells. Further investigations using more physiologically relevant systems are warranted to explore the relevant mechanisms. Second, under the current experimental conditions, an unambiguous distinction between nuclear and cytoplasmic localization of PPARα and PPARγ in HaCaT cells could not be established from the immunofluorescence data. Finally, the transcriptomic data were generated exclusively using the AOU and XASO treatments, rather than the AGX mixture itself, and the key differentially expressed genes identified by RNA-seq were not further validated by RT-qPCR. Consequently, any mechanistic interpretation regarding AGX remains an indirect inference, necessitating validation through future direct profiling. To address these limitations, subsequent studies should focus on elucidating the specific upstream and downstream regulatory relationships within the gene network and employ more advanced imaging techniques, such as confocal microscopy, to precisely define the subcellular localization of PPARα/γ.

## 5 Conclusions

In this study, we demonstrated the AGX, a complex derived from AOU, GLSO and XASO, has the potential to enhance skin barrier repair functionality and improve barrier homeostasis as a dual activator of PPARα and PPARγ. This research contributes to the growing knowledge relating to PPAR activation in skin health and provides a basis for future investigations and clinical applications.

## Supplementary Material

Supplementary Material
