# Supplementary material for: Plant oil complex: dual activation of peroxisome proliferator-activated receptors α and γ to enhance skin barrier function
Source: Open Life Sci. 2026 Jul 7;21(1):20251347. doi: 10.1515/biol-2025-1347 (PMC13340833; doi:10.1515/biol-2025-1347)
Supplement: Supplementary file 1 — Supplementary Material [file j_biol-2025-1347_suppl_001.docx]

**Supplementary material**

**1.1 Volunteer profiles, treatment methods and process the human study**

Sixteen healthy volunteers (4 males, 12 females) aged 25-38 years (mean: 31.06 ± 1.023). Volunteers without skin lesions, diagnosed with diabetes, or taking non-steroidal anti-inflammatory drugs were excluded. Subjects were allowed to touch water (shower/bath) as usual but were instructed to avoid the direct application of detergents, moisturizers, emollients, or topical medications to their forearms seven days before and during the study period. They were also asked to avoid activities that could induce strong sweating (e.g., sports) during the study period.

The volar forearm, with a relatively low density of hair follicles and little influence from daily activities, was selected as the test site. Five square test areas, each measuring 2 cm × 2 cm and spaced 1 cm apart, were marked on the volar forearm of each subject and irritated with an 8 mm diameter Finn Chamber (SmartPractice®, USA) containing 1% SLS; subsequently, the test area was covered for 24 h. Following three days without treatment, we measured the TEWL value and water content of the stratum corneum. Then, except for the blank group, the remaining four test areas received treatment as follows. Two samples (the AGX plant oil complex at concentrations of 2% and 4%), the placebo (Caprylic/Capric Triglyceride, GTCC) and a positive control drug (Dexamethasone Acetate Cream) were applied twice a day for seven consecutive days. Samples (13 μL) of each test solution were applied to each test area with a pipette, and spread evenly spread over the test area with the same manipulation and force. Notably, the test areas for each subject were randomly assigned to eliminate potential influences of individual skin differences on the experiment.

For each test day, it was necessary to adapt to a constant temperature and humidity (room temperature: 21 ± 1 ℃; relative humidity: 50 ± 10%) for 30 min. Evaluation of the skin barrier was performed with bioengineering methods (TEWL; Tewameter TM Hex; Courage & Khazaka, Cologne, Germany) and stratum corneum hydration (Corneometer CM 825; Courage & Khazaka, Cologne, Germany). All measurements were repeated three times for each subject area and the mean value was taken for analysis.
